# Gastric metastasis of renal cell carcinoma treated with endoscopic resection: A case report

**DOI:** 10.1002/ccr3.9076

**Published:** 2024-06-14

**Authors:** Kaori Yamashita, Satoshi Kubota, Harutoshi Sugiyama, Keita Yoshida, Takahiro Shiseki, Tetsushi Sakamoto, Tadao Nakazawa, Masashi Inui

**Affiliations:** ^1^ Department of Urology Tokyo Women's Medical University Yachiyo Medical Center Chiba Japan; ^2^ Department of Gastroenterology Tokyo Women's Medical University Yachiyo Medical Center Chiba Japan; ^3^ Department of Pathology Tokyo Women's Medical University Yachiyo Medical Center Chiba Japan; ^4^ Department of Urology, Graduate School of Medicine Chiba University Chiba Japan

**Keywords:** cancer, kidney, oncology, stomach

## Abstract

Gastric metastasis of renal cell carcinoma (RCC) is rarely encountered. The time interval between the primary diagnosis of RCC and the occurrence of gastric metastasis tends to occur after more than 10 years. Clinicians should be diligent in checking the general symptoms of patients for more than 10 years.

## 1 INTRODUCTION

Gastric metastasis of renal cell carcinoma (RCC) is very rare. Common metastatic sites of RCC are the lungs, bone, nonregional lymph nodes, ipsilateral adrenal gland, contralateral adrenal gland, pancreas, contralateral retroperitoneum, and liver.^1^

However, the time interval between the primary diagnosis of RCC and the occurrence of gastric metastasis is more than 10 years.^2^, ^3^, ^4^, ^5^, ^6^, ^7^ The treatment of gastric metastasis of RCC requires endoscopy,^2^, ^3^, ^5^, ^7^, ^8^, ^9^, ^10^, ^11^, ^12^ laparoscopy,^4^, ^13^, ^14^ total gastrectomy,^15^ radiation,^6^, ^9^ or chemotherapy.^3^, ^16^

We report a patient with gastric metastasis of RCC who was treated with endoscopic resection. Furthermore, we reviewed the recent literature on the clinical assessment, and the management of cases of gastric metastasis of RCC.

## 2 CASE HISTORY/EXAMINATION

In December 2016 during a health examination, a 77‐year‐old Japanese man presented with a left kidney tumor on computed tomography (CT). He had no symptoms and his physical examination was intact at that time. CT revealed a solid renal tumor with enhancement and a left kidney diameter of 45 mm, but no metastasis. In April 2017, he underwent laparoscopic radical nephrectomy. The histopathological diagnosis was clear cell RCC (ccRCC), pT1a, INFa, ly0, v0. In April 2022, CT revealed 6 and 3‐mm diameter metastases in the left lung, and thoracoscopic resection was performed. Pathology results showed metastasis of ccRCC. No adjuvant chemotherapy was administered because of the patient's advanced age. The patient was followed up.

In May 2023, which was 6 years after the primary RCC diagnosis, the patient, who was now 83 years old, visited our hospital complaining of melena and dizziness.

## 3 DIFFERENTIAL DIAGNOSES/INVESTIGATION/TREATMENT

### 3.1 Differential diagnoses

Differential diagnoses include gastroduodenal ulcer, gastroesophageal variceal rupture, Mallory–Weiss syndrome, small bowel bleeding, esophageal cancer, and stomach cancer.

### 3.2 Investigations

**FIGURE 1:**
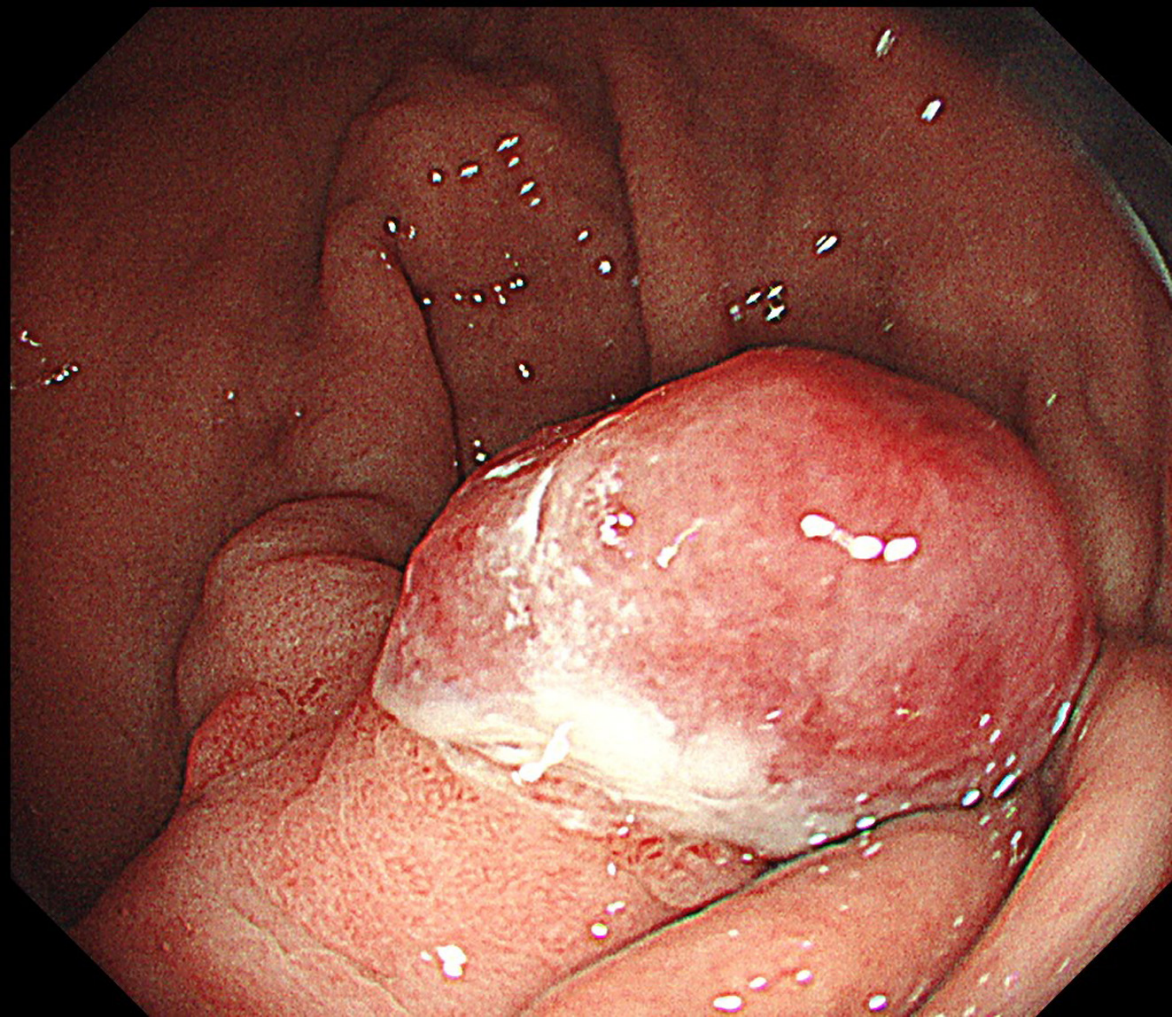
Imaging findings of gastrointestinal endoscopy. The gastric tumor is a mass with diameter of 15 mm in the body of the stomach.

**FIGURE 2:**
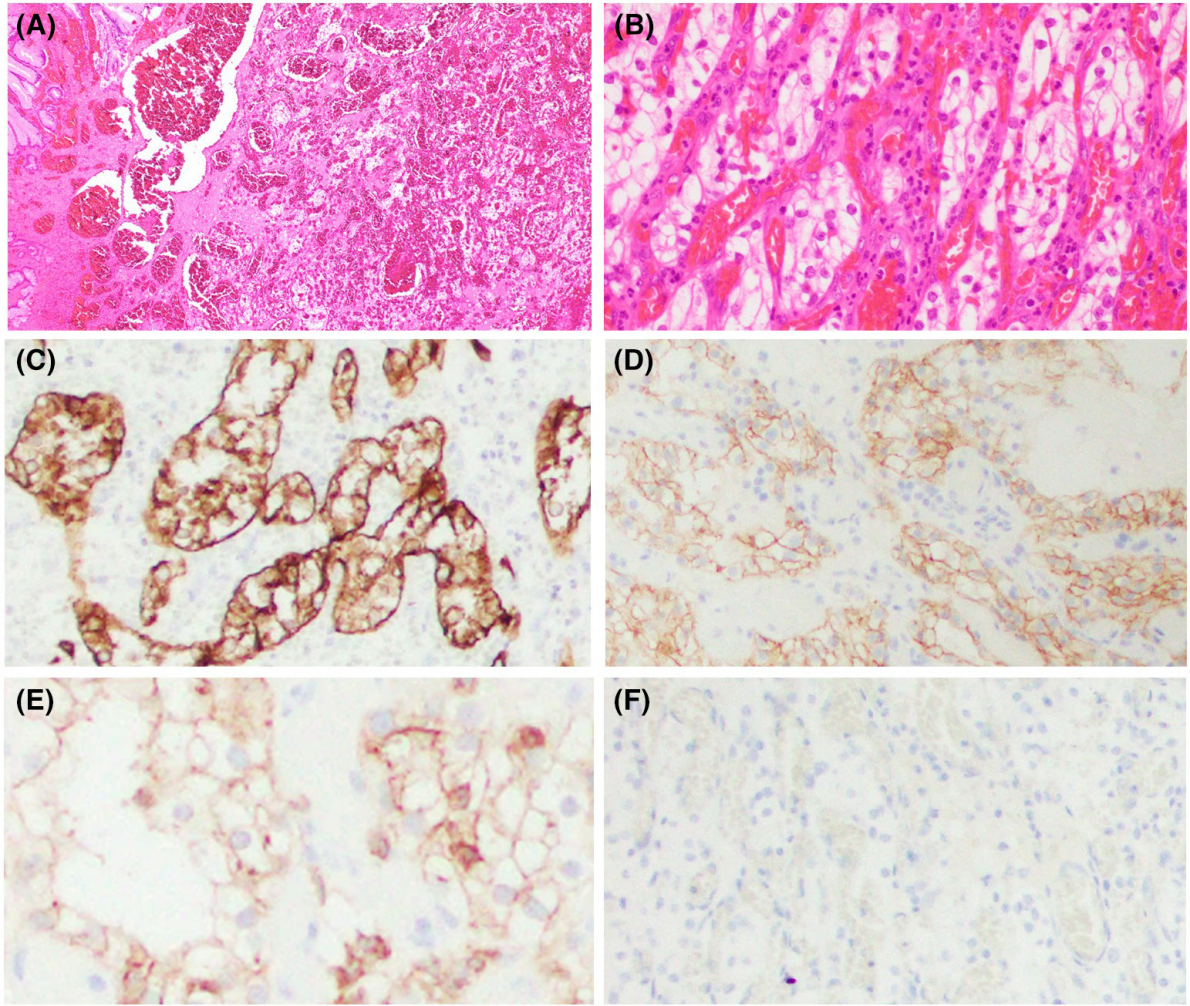
Microscopic findings and immunohistochemistry results. (A) The tumor is composed of nests circumscribed by abundant vascular stroma with hemorrhage. (B) Neoplastic cells show nuclear atypia of grade 2 and clear cytoplasm under high‐power magnification (H&E; magnification, ×40). (C–F) The membrane of carcinoma cells exhibits immunopositivity for CAM 5.2, CA9, and CD10 (C: CAM5.2; D: CA9; E: CD10) and are devoid of reactivity for CK7 (F). CA9, carbonic anhydrase 9; CD10, cluster of differentiation 10; CK7, cytokeratin 7; H&E, hematoxylin and eosin.

Blood test results showed a Hb level of 6.7 g/dL and blood urea nitrogen level of 55.9 mg/dL. Gastrointestinal endoscopy revealed a 15 mm tumor in the body of the stomach (Figure 1). CT did not show a tumor in the stomach. Endoscopic mucosal resection was performed. The polypectomy specimen from the stomach was routinely processed and embedded in a formalin‐fixed paraffin‐embedded block. The microscopic examination revealed that polygonal or round cells with clear cytoplasm formed nests and infiltrated throughout the submucosa to the tissue (Figure 2A). Sinusoid‐like capillary vessels were interspersed among the tumor nests and were filled with red blood cells with hemorrhage. These findings are consistent with metastasis of ccRCC to the stomach. According to the World Health Organization/International Society of Urological Pathology grading system, the neoplastic cells harbored grade 2 nuclear features, based on characteristics such as enlarged nuclei, and small nucleoli (Figure 2B). Immunohistochemical results revealed the typical labeling pattern of ccRCC. Carcinoma cells showed positive immunoreactivity for carbonic anhydrase 9 (CA9), cluster of differentiation 10 (CD10), and CAM5.2, but were entirely negative for cytokeratin 7 (CK7) (Figure 2C–F). The margin of this specimen was pathologically positive.

### 3.3 Treatment

We were hesitant to use immunotherapy because of the patient's advanced age and the occurrence of immune‐related adverse events. We consulted a gastrointestinal surgeon, who indicated the need for partial gastrectomy because the margin of endoscopic mucosal resection was pathologically positive. Pathological specimens from partial gastrectomy had no residual carcinoma cells and negative surgical margins.

## 4 OUTCOME/FOLLOW‐UP

During 6 months of follow‐up after the partial gastrectomy, the patient lived in good health with no adjuvant therapy, and CT revealed no recurrence or metastasis of RCC.

## 5 DISCUSSION

RCC is the 14th most common malignancy globally, according to the Global Cancer Observatory.^17^ Lyon et al.^1^ summarized the anatomical sites of 740 metastasectomies of RCC and reported that the metastatic sites of RCC are the lungs (36%), bone (13%), nonregional lymph node (9%), ipsilateral adrenal gland (8%), contralateral adrenal gland (8%), pancreas (8%), contralateral retroperitoneum (5%), and liver (3%), whereas gastric metastasis of RCC is uncommon. Some clinical case reports with gastric metastasis of RCC have been published, based on our literature investigation.^2^, ^3^, ^4^, ^5^, ^6^, ^7^, ^8^, ^9^, ^10^, ^11^, ^12^, ^13^, ^14^, ^15^, ^16^ We summarized a literature review of 16 case reports of gastric metastasis of RCC that were conducted in recent years (Table 1). The pathology of all cases of RCC was ccRCC, except one case for which the pathology was not reported. The average time interval between the primary diagnosis of RCC and the occurrence of gastric metastasis was 10.6 years (with a range of 0–28 years), and gastric metastasis occurred more than 10 years after the primary RCC in 7 of 16 cases. In 11 of the 16 cases, metastasis to other organs such as lung or bone occurred before gastric metastasis. Table 2 shows that, among symptoms of gastric metastasis such as melena or upper gastrointestinal bleeding, melena was the most common presentation as a gastrointestinal symptom: it occurred in 8 of the 16 cases. However, systemic symptoms of general malaise, dizziness, anorexia, or anemia were also present. We recommend that urologists check RCC patients' general symptoms and blood tests for more than 10 years.

**TABLE 1:** Summary of reported clinical cases of RCC with gastric metastasis.

First author (publishing year)	Age, year (gastric metastasis)/sex	Treatment for primary tumor	Primary stage/pathology	Metastasis to other organs			Gastric metastasis		Outcomes after treatment of gastric metastasis
				Location of metastasis	Time interval to metastasis to other organs (year)	Adjuvant therapy for metastasis to other organs	Time interval to gastric metastasis (year)	Treatment of gastric metastasis	
Michigami et al.^2^ (2023)	81/F	RN	Unknown/ccRCC	Pancreas	4	Pancreatectomy	10	Endoscopy	3‐month survival
Cano et al.^4^ (2023)	77/M	RN	T2N0M0/ccRCC	Bone	4	Sunitinib	22	Atypical gastrectomy by laparotomy	Unknown
				Abdominal wall	8				
Degerli et al.^3^ (2022)	73/M	RN	T2bN0M0/ccRCC	Lung, L/N	7	Sunitinib	14	Endoscopy, sunitinib	Unknown
Kim et al.^8^ (2022)	70/F	RN	T1bN0M0/ccRCC	Contralateral kidney	4	Cryoablation	7	Endoscopy	Recurrence at the gastric body after 3 months
				bone	6				
Chen et al.^10^ (2022)	65/M	RN	Unknown/ccRCC	Gallbladder, Pancreas, Soft tissue	5	Sunitinib, axitinib	5	Endoscopy	1‐year survival
Mcllwaine et al.^5^ (2022)	80/F	RN	Unknown/ccRCC	Lung	21	Refused further investigation	21	Endoscopy	5‐year survival
Tapasak et al.^15^ (2022)	77/M	RN	T3aNxM1/ccRCC	Diaphragm	0	Chemotherapy	5	Endoscopy, total gastrectomy	Unknown
Hakim et al.^9^ (2021)	86/F	RN	Unknown/ccRCC	Contralateral kidney	12	PN	28	Endoscopy, Radiation	Unknown
				Bone, lung, L/N	18				
Parmar et al.^16^ (2021)	65/M	CN	TxN0M1/ccRCC	None			Synchronous	Gastric SOL excision, pazopanib	1‐year survival
Prudhomme et al.^13^ (2021)	61/M	PN	T3aN0M0/ccRCC	Homolateral kidney	2	RN	8	Laparotomy	2‐year survival
Koterazawa et al.^11^ (2021)	70/F	RN	T3aN0M1/ccRCC	None			Synchronous	Endoscopy	4‐month survival
Orosz et al.^6^ (2021)	73/M	RN	Unknown/ccRCC	Brain	20	None	20	Acid suppression, radiation	Unknown
Bernshteyn et al.^12^ (2020)	68/M	RN	Stage4/unknown	Skin, lung	Synchronous	None	Synchronous	Endoscopy	Unknown
Yoshida et al.^7^ (2020)	85/F	RN	T2N0M0/ccRCC	None			15	Endoscopy	2‐year survival
Kinoshita et al.^14^ (2019)	60/M	RN	T1bN0M0/ccRCC	None			3	Laparoscopic and endoscopic cooperative surgery	1‐year survival
Our case	83	RN	T1aN0M0/ccRCC	Lung	5	Thoracoscopic resection	6	Endoscopy	4‐month survival

Abbreviations: ccRCC, clear cell renal cell carcinoma; CN, cytoreductive nephrectomy; L/N, lymph node; M, male; F, female; PN, partial nephrectomy; RCC, renal cell carcinoma; RN, radical nephrectomy; SOL, solitary space‐occupying lesion.

**TABLE 2:** Symptoms of gastric metastasis.

	Cases (*N*)
Gastrointestinal symptoms	
Melena	8
Upper gastrointestinal bleeding	4
Abdominal discomfort	2
Systemic symptoms	
General malaise	4
Dizziness	3
Anorexia	3
Anemia	2
Weight loss	2
Syncope	1
Pyrexia	1
Other	
Mass at routine computed tomography	1
Mass at routine gastrointestinal endoscopy	1

With regard to treatment, the gastric metastasis was removed successfully via gastrointestinal endoscopy in 8 of the 16 patients (including our patient).^2^, ^5^, ^7^, ^8^, ^11^, ^12^, ^14^ Five patients needed invasive surgeries such as laparoscopic or open gastrectomy.^4^, ^13^, ^14^, ^15^, ^16^ Other patients required radiation therapy^6^, ^9^ or molecular target drug therapy.^3^, ^16^ A gastric tumor can supposedly be resected via gastrointestinal endoscopy only, if clinicians detect a gastric metastasis in the early stage. If the margin of endoscopic mucosal resection in this patient had been pathologically negative, partial gastrectomy would not have been necessary. The line of treatment for metastatic RCC is immunotherapy (Check mate 214,^18^ Checkmate 9ER,^19^ and Keynote 426^20^). However, we were hesitant to use immunotherapy because of the patient's advanced age and the occurrence of immune‐related adverse events. In addition, if immunotherapy had been prescribed, partial gastrectomy would not have been necessary.

With regard to the prognosis of postoperative gastric metastasis of patients with RCC, 9 of the 16 patients (including our patient) had a survival of 3 months–5 years^2^, ^5^, ^7^, ^10^, ^11^, ^13^, ^14^, ^16^; only one patient had a recurrence in the gastric body 3 months after endoscopy for gastric metastasis of RCC.^8^

In conclusion, gastric metastasis of RCC is rarely encountered. The time interval between the primary diagnosis of RCC and the occurrence of gastric metastasis tends to occur after more than 10 years. Urologists should be diligent in checking the gastrointestinal symptoms and general symptoms of patients with RCC for more than 10 years to detect the early stage of gastric metastasis of RCC.

## AUTHOR CONTRIBUTIONS

**Kaori Yamashita:** Conceptualization; writing – original draft; writing – review and editing. **Satoshi Kubota:** Data curation. **Harutoshi Sugiyama:** Supervision. **Keita Yoshida:** Visualization; writing – original draft. **Takahiro Shiseki:** Data curation. **Tetsushi Sakamoto:** Data curation. **Tadao Nakazawa:** Supervision; writing – original draft. **Masashi Inui:** Supervision; writing – original draft.

## CONFLICT OF INTEREST STATEMENT

The authors have no conflicts of interest to declare.

## CONSENT

Written informed consent was obtained from the patient to publish this report in accordance with the journal's patient consent policy.

## Data Availability

The data that support the findings of this study are available from the corresponding author upon reasonable request.
